# Supplementary material for: EZH2-TTP-mTORC1 Axis Drives Phenotypic Plasticity and Therapeutic Vulnerability in Lethal Prostate Cancer
Source: Res Sq. 2025 Aug 21:rs.3.rs-7360528. Preprint. [Version 1] doi: 10.21203/rs.3.rs-7360528/v1 (PMC12393502; doi:10.21203/rs.3.rs-7360528/v1)
Supplement: 1 [file NIHPPRS7360528V1-supplement-1.pdf]

Supplementary Figure 1

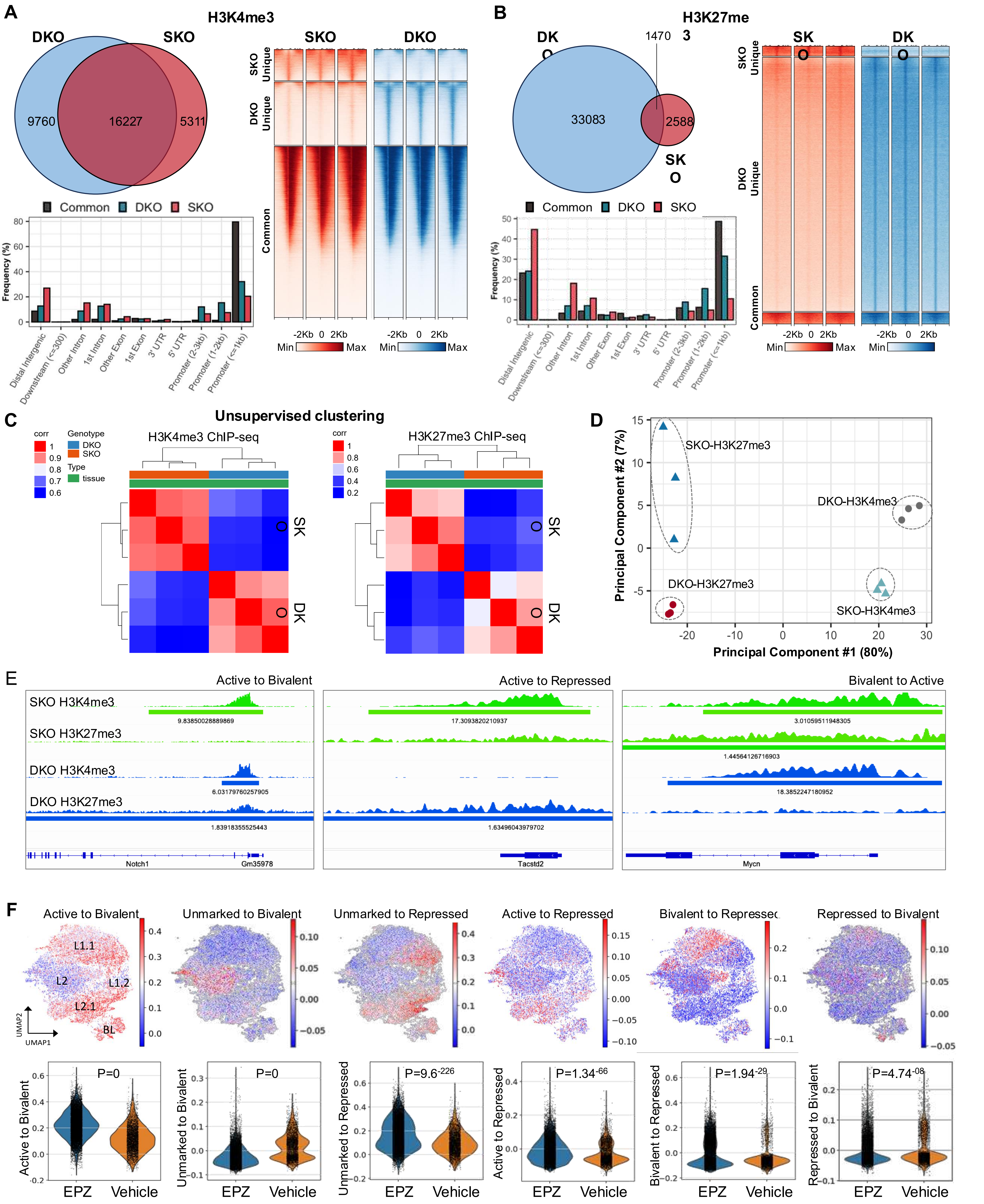

Supplementary figure 1:

EZH2 coordinates multi-lineage transitional cell states mediated by RB1 loss. **A**. Venn diagram (left) and tornado plot (right) illustrating the 31,298 ChIP-seq peaks for H3K4me3 in *PbCre:Pten KO* (SKO) and *PbCre:Pten:Rb1 KO* (DKO) tumors. Bar plot (bottom left) indicates H3K4me3 peak genome-wide peak distribution. 52% of the peaks were shared between SKO and DKO, 17% and 31% were considered unique in SKO and DKO respectively. The analysis of exact genomic location of H3K4me3 is illustrated in the bars plot and showed that most of the genomic locations are at promoter regions. **B.** Venn diagram (left) and tornado plot (right) illustrating the 37,141 ChIP-seq peaks for H3K27me3 in SKO and DKO tumors. 4% of the peaks were shared between SKO and DKO, 7% and 89% were considered unique in SKO and DKO respectively. The analysis of exact genomic location of H3K27me3 is illustrated in the bars plot and showed that most of the genomic locations are at promoter regions. **C.** Hierarchical clustering of SKO and DKO tumors based on sample-to-sample correlation of H3K4me3 and H3K27me3 peaks representing promoter occupancy. **D.** Two-dimensional principal component analysis (PCA) of H3K4me3 and H3K27me3 peaks representing promoter occupancy **E.** Gene track examples chromatin remodeling in Notch1 and Tacstd2 which move from an active to bivalent or repressed state respectively and Mycn from a bivalent to active state. **F.** UMAP representing the genes transitioning from Active to Bivalent, Unmarked to Bivalent, unmarked to repressed, active to repressed, bivalent to repressed and repressed to bivalent from SKO to DKO tumors based on the H3K4me3 and H3K27me3 ChIP-seq analysis (top). Violin plots of significantly enriched gene sets in DKO upon vehicle or EPZ treatment. FDR < 0.01, abs (NES) > 1 where NES is the normalized enrichment score.

Supplementary Figure 2

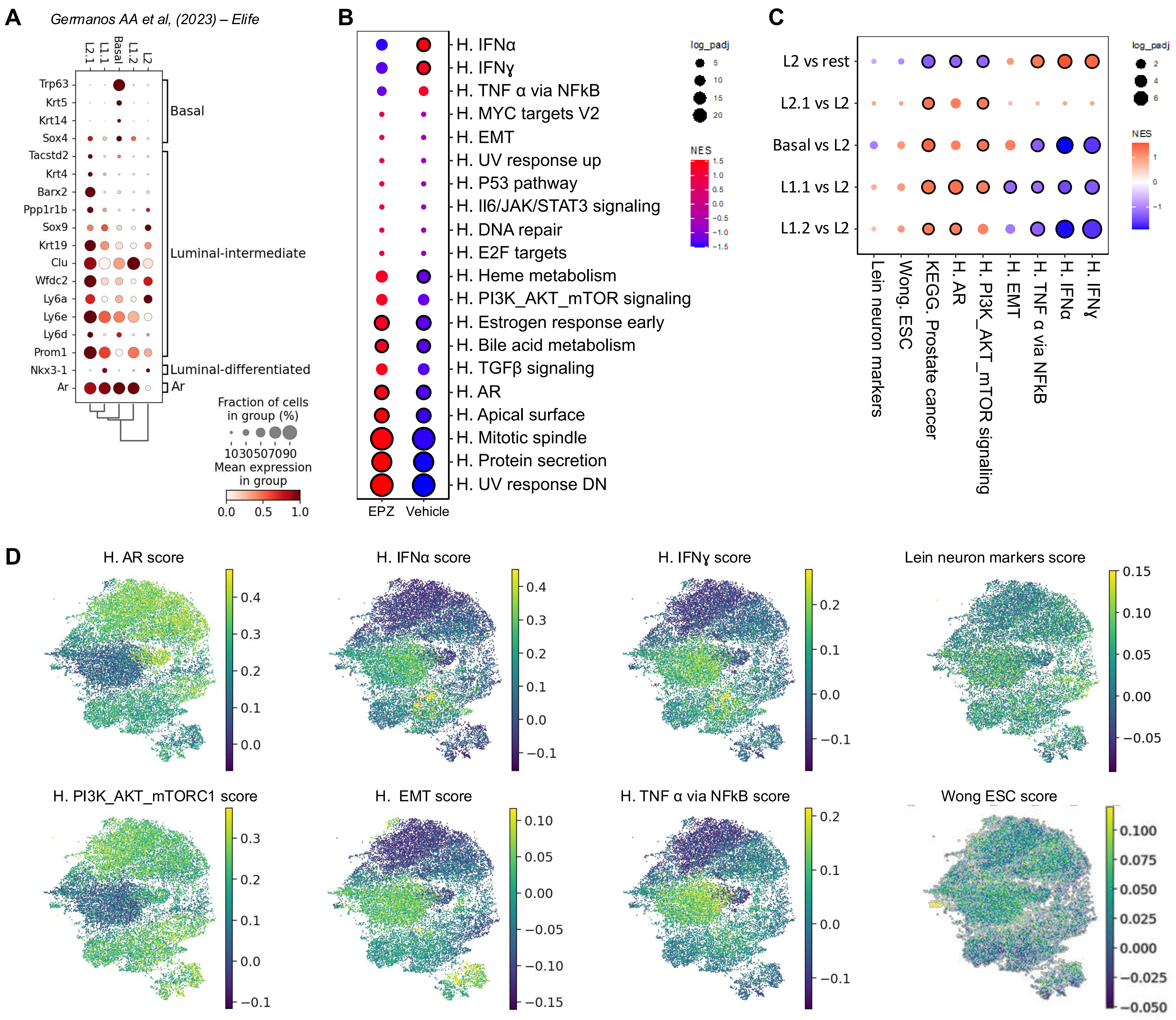

Supplementary Figure 2:

A. Dot plot of canonical marker genes for each cell type. Hue of red indicates average normalized gene expression level and size of dot indicates percentage of nonzero gene expression for each cell population. **B.** Dot plot of the Gene Set Enrichment Analysis (GSEA) of transcripts upregulated in EPZ treated mice compared with vehicle. **C.** Dot plot of the GSEA of transcripts upregulated in each epithelial sub-cluster compared with L2. **D.** UMAP of the androgen signaling (NELSON_ANDROGEN_ SIGNALING_UP), epithelial mesenchymal transition (EMT), interferon alpha response (IFNα), interferon gamma response (IFNɣ), PI3K-AKT-MTOR signaling and TNFα signaling via NFkB, embryonic stem cells.

Supplementary Figure 3

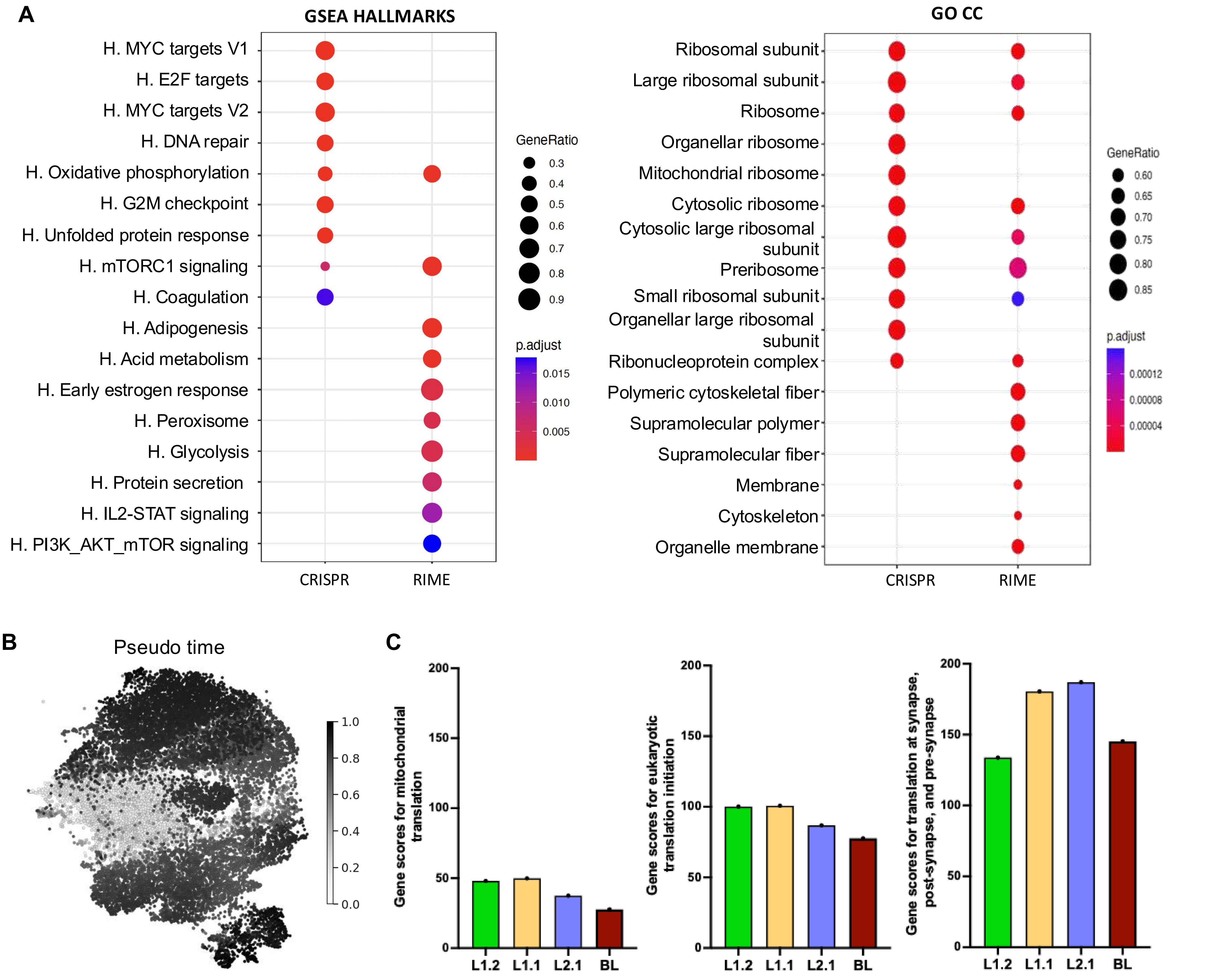

Supplementary Figure 3:
A. Dot plot representing the Gene Set Enrichment Analysis (GSEA) Hallmarks pathway and gene ontology cellular component analysis demonstrate overlay of common pathways and process's from Crispr screening and RIME analysis for DKO and SKO cells. The blue and red color represents the p-values across all cells, and the size of the dot corresponds to the percentage of cells expressing the characteristic for each pathway. **B.** Pseudo time analysis of epithelial cells in vehicle and EPZ treated mice. **C.** Bar plots showing the signature related with translation mechanisms in L2 sub-cluster versus other sub-clusters (L2.1, L1.1, L1.2 and BL). The analyzed signatures include genes related with processing of capped intron pre-mRNA, eukaryotic translation initiation, and mitochondrial translation.

Supplementary Figure 4

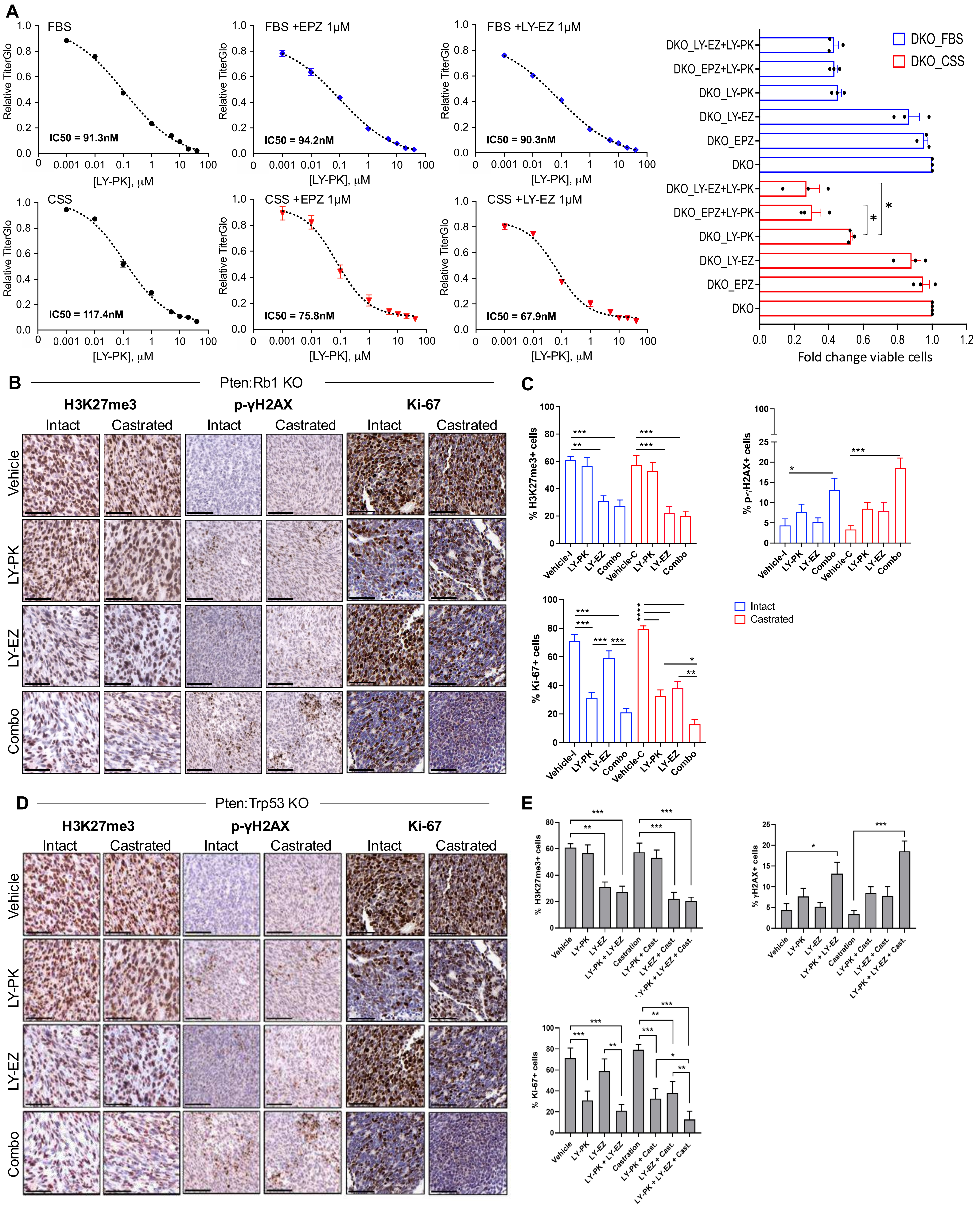

Supplementary figure 4:

A. Titre glo and cell viability fold change from DKO spheroids cultures in FBS and CSS with a LY-PK dose curve and 1µM of EPZ or LY-EZ. **B-E.** H3K27me3, p-□H2AX and Ki-67 IHC staining in murine DKO and PPKO tumors from the in vivo study, and the corresponding quantification of the percentage of positive cells (n = 5 mice per treatment group, +/-1SD).

Supplementary Figure 5

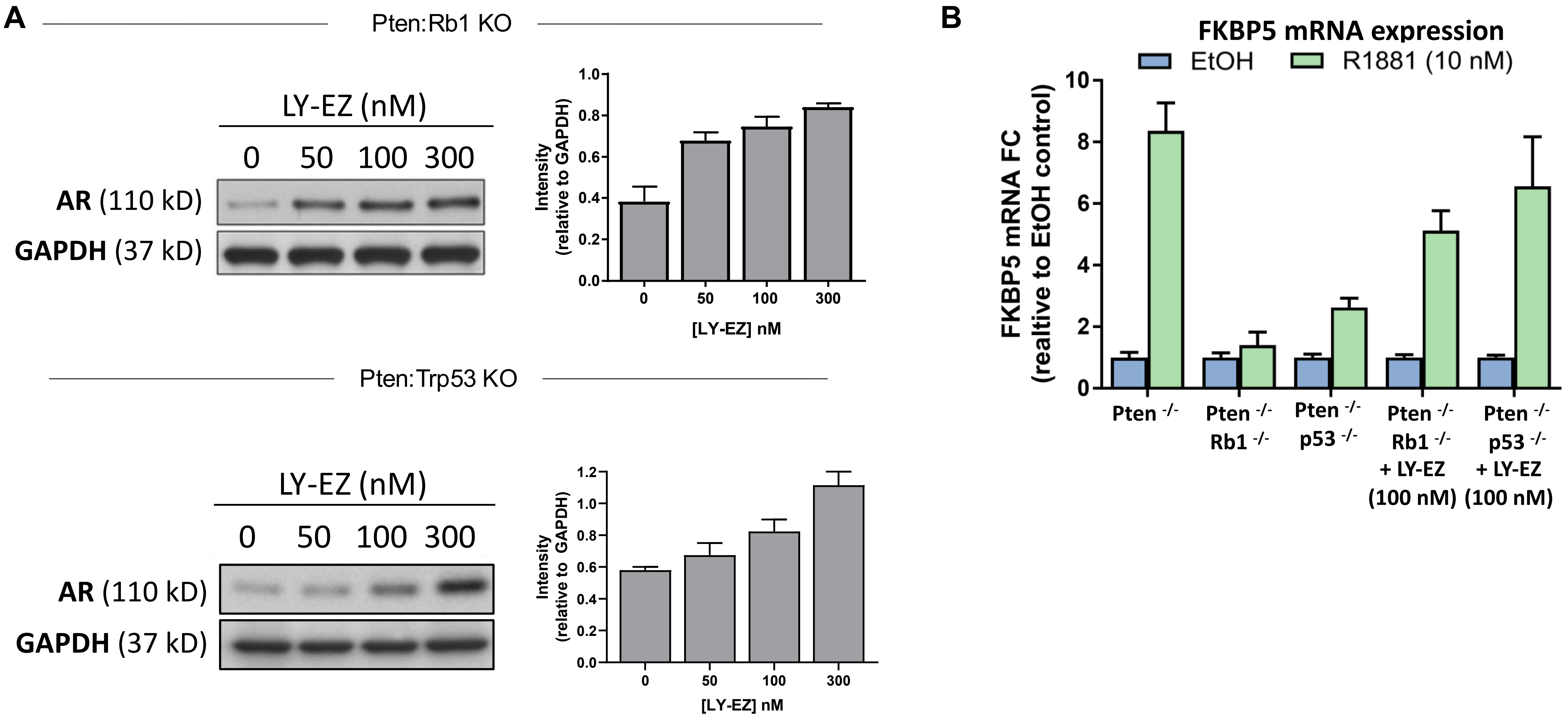

Supplementary figure 5:
A. Western blot indicating AR protein levels and quantification in the DKO and PPKO cells treated with DMSO or LY-EZ at different concentrations (indicated in the figure). **B.** Fold change of Fkbp5 in GEMM-derived DKO and PPKO cells treated with EtOH or R1881 (10 nM) stimulation for 24hrs, in triplicates.

Supplementary Figure 6

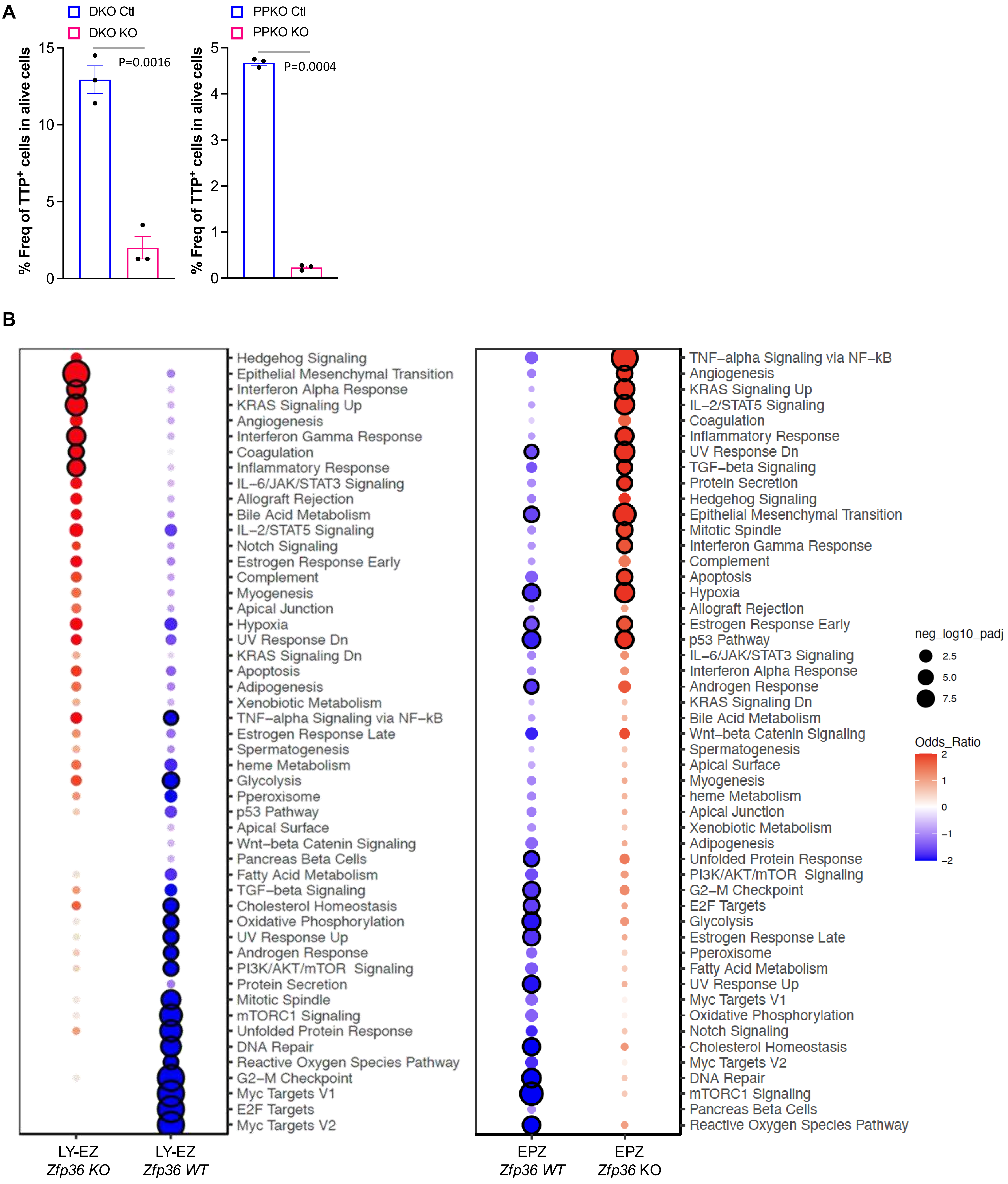

Supplementary figure 6:

A. Bar plot representing the TTP flow cytometry analysis in DKO Ctl, PPKO Ctl, DKO *Zfp36* KO, PPKO *Zfp36* KO. **B.** Extended bubble plot demonstrating GSEA analysis of indicated gene signatures from RNAseq derived from DKO and cells treated with DMSO controls or the EZH2i (EPZ6438 or LY-EZ, 5□M) for 96 hours. Hue of red indicates average normalized gene signature level and size of dot indicates percentage of nonzero gene signature.
